# Enlarged Abdominal Lymph Node as a Cause of Polyhydramnios in the Course of Congenital Neonatal Leukaemia: A Case Report and Review of the Literature on Foetal Abdominal Tumours with Coexisting Polyhydramnios

**DOI:** 10.3390/jcm11216598

**Published:** 2022-11-07

**Authors:** Daria Salloum, Paweł Jan Stanirowski, Aleksandra Symonides, Paweł Krajewski, Dorota Bomba-Opoń, Mirosław Wielgoś

**Affiliations:** 1st Department of Obstetrics and Gynecology, Medical University of Warsaw, Starynkiewicza Sq. 1/3, 02-015 Warsaw, Poland

**Keywords:** polyhydramnios, congenital neonatal leukaemia, abdominal tumour, lymph node

## Abstract

Polyhydramnios represents a complication found in 0.2–2% of pregnancies, and it is usually diagnosed between 31 and 36 weeks of pregnancy. Although most cases of polyhydramnios are idiopathic, maternal diabetes or foetal malformations constitute frequent causes of the excessive accumulation of the amniotic fluid. Considering the latter, polyhydramnios may rarely be caused by foetal abdominal tumours, with the incidence rate of 2–14 cases per 100,000 live births. Congenital neonatal leukaemia (CNL) is a rare disease with a reported incidence rate of 5–8.6 cases per million live births. In the prenatal period, the ultrasound abnormalities associated with CNL include hepatomegaly and splenomegaly. In this paper, we presented a case of polyhydramnios caused by mechanical pressure on the foetal gastrointestinal tract by an enlarged lymph node in the course of CNL, as well as reviewing the available literature on foetal abdominal tumours with concurrent polyhydramnios.

## 1. Introduction

Polyhydramnios represents a complication found in 0.2–2% of pregnancies, and it is usually diagnosed between 31 and 36 weeks of pregnancy [1]. Although most cases of polyhydramnios are idiopathic, maternal diabetes or foetal malformations constitute frequent causes of the excessive accumulation of the amniotic fluid [2]. Considering the latter, polyhydramnios may rarely be caused by foetal abdominal tumours, with the incidence rate of 2–14 cases per 100,000 live births [3]. The abdominal tumours most frequently diagnosed in the prenatal period include teratomas, neuroblastomas and hepatic tumours [4]. The mechanism responsible for the occurrence of polyhydramnios in the case of abdominal tumours may be twofold—the mechanical pressure on the gastrointestinal tract or hyperdynamic circulation leading to excessive foetal urination. Interestingly, the vast majority of described cases of abdominal tumours coexisting with excessive amniotic fluid volume concern solid, neoplastic lesions, and no case of polyhydramnios caused by reactive tumours in the course of myeloproliferative disorders has been published to date.

Congenital neonatal leukaemia (CNL) is a rare disease with a reported incidence rate of 5–8.6 cases per million live births [5]. The characteristic changes found in a neonate include abnormal blood count (hyperleukocytosis, thrombocytopenia and anaemia), enlargement of internal organs, such as liver and spleen, and skin infiltration [6,7]. In the prenatal period, the ultrasound abnormalities associated with CNL include hepatomegaly and splenomegaly, as well as generalised foetal oedema [8,9]. The prognosis in most cases of CNL is unfavourable, with a 23% survival rate at 24 months [10].

This paper presents a review of the literature on foetal abdominal tumours coexisting with polyhydramnios. The analysis is based on a rare case of polyhydramnios diagnosed in the third trimester of pregnancy and caused by mechanical pressure on the gastrointestinal tract by an enlarged lymph node in the course of CNL. The above-mentioned observation emphasizes the need to differentiate between abdominal tumours of different aetiology, including myeloproliferative neoplasms, as potential causes of abnormal amniotic fluid volume.

## 2. Materials and Methods

A review of the English and Polish literature was undertaken for articles published between January 1980 and September 2022 to identify case reports and case series related to foetal abdominal tumours coexisting with polyhydramnios. Studies were identified via PubMed, Scopus, EMBASE and Web of Science database searching using the key words: “polyhydramnios”; “abdominal tumour” and “congenital neonatal leukaemia” by two authors independently (DS, PJS). The reference lists of retrieved articles were reviewed to locate additional studies. Reviews and articles written in language other than English and Polish, as well as cases of foetal tumours without concomitant polyhydramnios, were excluded from further analysis.

After the initial literature search, publications were analysed by title and abstract to exclude studies that did not meet the inclusion criteria. Following abstract selection, the remaining full-text articles were screened for eligibility. The following data were collected by two investigators independently: tumour type, gestational age at diagnosis, ultrasound findings, pregnancy management and neonatal outcome.

## 3. Case Presentation

A 38-year-old patient (gravida VII, para IV) was admitted to the clinic at 34 + 6 gestational weeks due to the observed increase in the abdominal circumference for several days and dyspnoea. The course of the pregnancy was uncomplicated so far, and the only maternal co-morbidity observed at the admission was moderate obesity (BMI 38 kg/m^2^). The result of a combined test performed at 12 weeks of pregnancy indicated a high risk of trisomy 21 (1:65). Nonetheless, the patient did not follow up with further diagnostics, including invasive procedures, as well as did not report for ultrasound examinations for the assessment of foetal anatomy between 18–22 and 28–32 weeks of pregnancy, as recommended by the Polish Society of Gynaecologists and Obstetricians [11].

**Figure 1:**
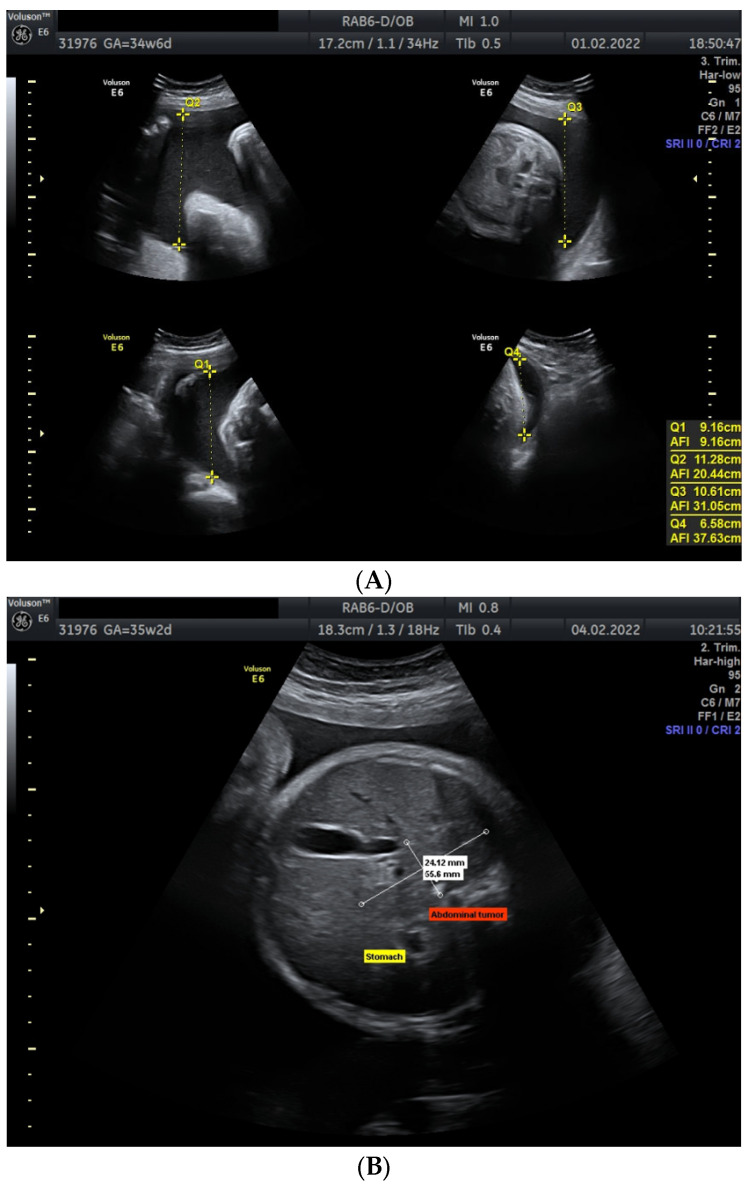

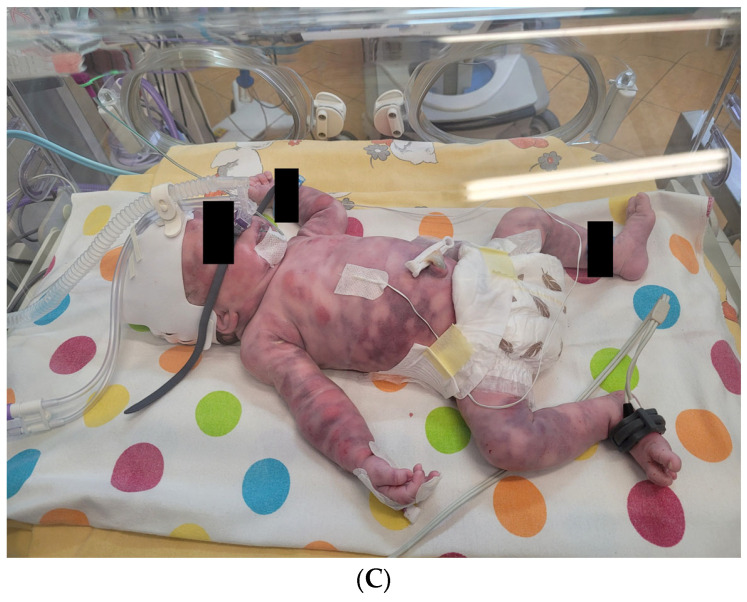
(**A**) Ultrasound image presenting amniotic fluid index measurement. (**B**) Ultrasound image presenting foetal abdomen and tumour in the hepatic hilum area. (**C**) Bruisings and nodular lesions on the skin of the neonate with congenital neonatal leukaemia.

The physical examination at admission did not reveal any abnormalities, both blood pressure and heart rate were normal. The CTG recording was reactive, with normal variability and a short-term variability of 8.7 ms. Laboratory tests revealed haemoglobin, leukocyte and platelet concentrations of 12.6 g/dL; 10,700/µL and 292,000/µL, respectively. The foetal ultrasound demonstrated severe polyhydramnios, with amniotic fluid index (AFI) of 37.63 cm, and small stomach (Figure 1A,B). Doppler indices in both the umbilical artery and middle cerebral artery were normal (MCA PSV 60.8 cm/s = 1.206 MoM). Due to difficulties in visualization associated with the patient’s obesity and polyhydramnios, a precise anatomy assessment of the foetus was not performed during initial ultrasound examination. Due to the excessive accumulation of amniotic fluid, resulting in clinical symptoms as well as high risk of chromosomal aberration in the foetus, an amnioreduction was performed following the patient’s consent, and amniotic fluid was collected for the determination of foetal karyotype (result: 46 XX). The procedure was uncomplicated, and 1600 mL of clear amniotic fluid was collected. After two days, the patient again reported dyspnoea. A second amnioreduction was performed, and 1700 mL of amniotic fluid was collected. Three days later, the last amnioreduction was conducted, resulting in the collection of 1700 mL of clear fluid. During the procedure, the ultrasound examination revealed the presence of a solid tumour in the hepatic hilum area, measuring 55.6 × 24.12 mm, without signs of increased vascularisation (Figure 1B). Due to the presence of the tumour, the patient was qualified for elective Caesarean section. At 35 + 6 gestational weeks a female foetus was born, weighing 3400 g, in moderately good general condition (6-6-6-7 Apgar points).

The physical examination of the neonate revealed numerous bruisings and nodular lesions on the skin (Figure 1C). Laboratory tests revealed thrombocytopenia (25,000 µ/L), leukocytosis (33,400 µ/L), anaemia (Hg 8.3 g/dL) and abnormalities in the coagulation profile (APTT 107 s, INR 4.59). A total of 98% of the cells found in the manual blood smear were blast cells. The ultrasound of the neonate’s abdomen confirmed presence of a tumour measuring 58.5 × 25.1 mm localized in the hepatic hilum area. Due to the severe general condition, subdural haemorrhage and suspected CNL, the neonate was transferred to the Department of Neonatology, Pathology and Intensive Therapy at the Children’s Memorial Health Institute. Two days after the birth, the neonate died. The histopathological examination of the neonate confirmed the diagnosis of a diffuse neoplastic process in the course of CNL and the presence of an enlarged abdominal lymph node. At the same time the histopathological examination of the placenta did not reveal any abnormalities.

## 4. Results and Discussion

**Figure 2:**
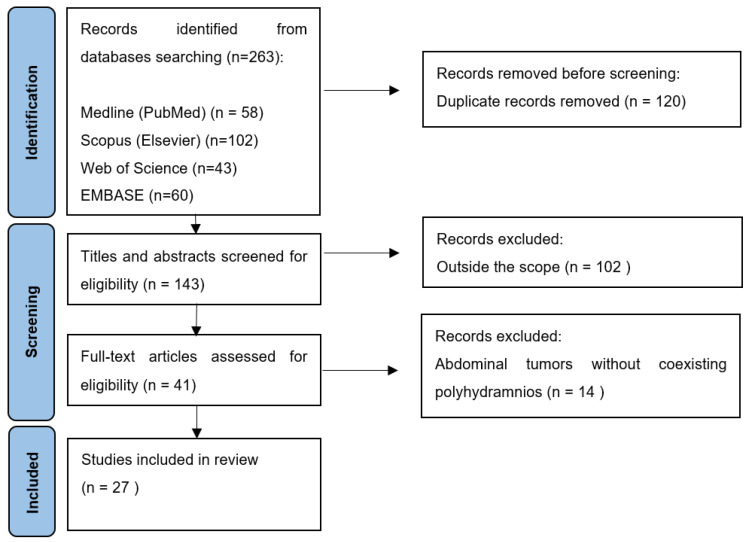
Flow chart displaying the selection process.

In this study we presented a case of polyhydramnios caused by mechanical pressure on the foetal gastrointestinal tract by an enlarged lymph node in the course of CNL, as well as reviewing the available literature on foetal abdominal tumours with concurrent polyhydramnios. A total of 263 articles were identified through a database search, of which 120 were duplicates (Figure 2). Following abstract screening, 102 articles were determined to be outside the scope of the investigation and a further 14 full-text articles described cases of abdominal tumours without concomitant polyhydramnios. The remaining 27 publications describing 32 cases constituted the basis of this review (Supplementary Table S1), [12,13,14,15,16,17,18,19,20,21,22,23,24,25,26,27,28,29,30,31,32,33,34,35,36,37,38].

In the prenatal period, leukaemia is found much less frequently than in childhood [39]. A higher risk of leukaemia has been identified in patients with Down syndrome [40]. In the presented case, despite a high risk of trisomy 21 in the combined test, we did not demonstrate any abnormalities in the foetal karyotype. Only one case of pregnancy complicated by polyhydramnios in the course of CNL was described in the available literature [41]. According to the authors’ suggestion, the excessive volume of the amniotic fluid could result from the neoplastic invasion of the placental villi, which was confirmed in their histopathological examination. In our case, however, no neoplastic lesions were found in the placental tissue, and the most probable cause of the polyhydramnios was the mechanical pressure on the gastrointestinal tract by an enlarged lymph node localized in the hepatic hilum area.

The analysis of the literature revealed that the mean gestational age in which an abdominal tumour coexisting with polyhydramnios was diagnosed is 34 weeks (18–36) (Supplementary Table S1). In 9 out of 32 cases (28.1%) an amnioreduction was performed, and in 8 cases (25%) the premature rupture of membranes occurred (in 4 cases following amnioreduction). The preferred mode of delivery was Caesarean section (62.5%; 20/32), including four cases (20%) classified as an emergency CS. The mean gestational age at the delivery was 35 weeks (25–40). In only one case a decision was made to terminate the pregnancy [37].

Half of the analysed cases of abdominal tumours concurrent with polyhydramnios were represented by mesoblastic nephroma (50%, 16/32), a rare tumour of mesenchymal origin, accounting for 3% of the renal tumours diagnosed in children [42]. The majority of mesoblastic nephroma cases (90%) are diagnosed in the first year of life [43]. Regarding the prenatal period, the mean gestational age at the diagnosis was 31 weeks (22–36). Importantly, in over half (9/16) of the ultrasound examinations a renal mass was detected. In 56% of cases a Caesarean section was performed, and the mean gestational age at delivery was 36 weeks (25–38). The preferred method of treatment of mesoblastic nephroma is surgery—depending on its histological type, only the tumour is excised, or nephrectomy or nephroureterectomy is conducted [44]. In most cases the prognosis is favourable [22]. In the analysed group of children, only two neonates died soon after birth, while in the remaining patients no disease recurrence was found during a follow-up period of 6 to 36 months.

In the available literature, the second most common abdominal tumour coexisting with polyhydramnios is immature gastric teratoma (9.4%, 3/32) (Supplementary Table S1). It is a very rare tumour, accounting for less than 2% of abdominal tumours diagnosed in neonates. In three reported cases of immature gastric teratoma, ultrasound studies revealed not only polyhydramnios, but also placentomegaly, ascites and intraperitoneal calcifications [34,35,36,45]. Treatment of a tumour in the post-partum period involves removal of the lesion, sometimes combined with a partial gastric resection. All the presented cases of pregnancies with immature gastric teratomas were delivered by Caesarean section.

Similarly to immature gastric teratoma, congenital neuroblastoma is a rare tumour, accounting for 5% of neuroblastomas diagnosed in children [46]. It originates from the neural crest cells which, in normal conditions, form sympathetic ganglia and adrenal medullas [47]. Due to the presence of characteristic “blueberry muffin” skin lesions in neonates, neuroblastoma is frequently considered in differential diagnosis of CNL [48]. Congenital neuroblastoma is most frequently diagnosed in the 3rd trimester of pregnancy, and apart from the presence of tumour lesions in the abdomen, the most common ultrasound findings include generalised foetal oedema, placentomegaly and placental metastases (Supplementary Table S1) [49]. The 10-year survival rate is approximately 49% [35], and the preferred method of treatment is chemotherapy [50]. In the analysed literature, pregnancies complicated by polyhydramnios and congenital neuroblastoma were delivered via Caesarean section, and the treatment method of choice was chemotherapy, as mentioned before.

Nephroblastoma, also known as Wilms’ tumour, is the most common kidney tumour diagnosed in children [51]. It is frequently associated with congenital defects or genetic syndromes, such as Beckwith–Wiedemann syndrome [35]. Ultrasound findings include foetal oedema, ascites and nephromegaly (Supplementary Table S1). The preferred method of treatment in most cases is laparotomy and nephrectomy, without the need for adjuvant chemotherapy [52]. Despite the favourable prognosis—the overall survival rate is approximately 90%—in the reported cases associated with polyhydramnios, one of the neonates died shortly after the delivery, while the other one demonstrated developmental delay one year after the delivery and laparotomy.

## 5. Conclusions

In the course of pregnancy, abdominal tumours constitute rare causes of polyhydramnios. During the ultrasound examination of excessive amniotic fluid volume, attention should be paid to potential abnormal masses in the foetal abdomen. In rare cases, the mechanical pressure on the foetal gastrointestinal tract may be caused not only by neoplastic tumours, but also by a reactive lymph node in the course of a myeloproliferative diseases.

## Data Availability

The data that support the findings of this study are available from the corresponding author on reasonable request. The data are not publicly available due to privacy or ethical restrictions.

## Supplementary Materials

The following supporting information can be downloaded at: https://www.mdpi.com/article/10.3390/jcm11216598/s1, Table S1: Foetal Abdominal Tumours with Coexisting Polyhydramnios.
