# Supplementary material for: Enlarged Abdominal Lymph Node as a Cause of Polyhydramnios in the Course of Congenital Neonatal Leukaemia: A Case Report and Review of the Literature on Foetal Abdominal Tumours with Coexisting Polyhydramnios
Source: J Clin Med. 2022 Nov 7;11(21):6598. doi: 10.3390/jcm11216598 (PMC9656261; doi:10.3390/jcm11216598)
Supplement: Supplementary file 1 [file jcm-11-06598-s001.zip › jcm-1969030-supplementary.pdf]

Table S1. Summary of studies included in the review.

No.	Author, year [reference number]	Number of cases	Tumour type	GA at diagnosis (weeks)	Ultrasound findings	Pregnancy management	Neonatal outcome
1.	Ben David et. al. 2021 [12]	1	granulosa cell tumour	30	intra-adominal mass, polyhydramnios, ascites, swelling of the genitalia	CS due to pPROM at 33+2 weeks	laparotomy 10 days following the delivery<br>3rd month after the delivery - remission
2.	Schurr Patti et al. 2008 [13]	1	infantile myofibroma	35	polyhydramnios, foetal ascites, diaphragmatic hernia	elective CS at 37 weeks	at birth: distended abdomen, prominent abdominal veins, hepatomegaly, bounding femoral pulses and edema.<br>63 days after the delivery discharged with nasogastric tube
3.	Küpeli, Serhan et al. 2015 [14]	1	neuroblastoma	3rd trimester (exact week unknown)	polyhydramnios, hyperechogenic mass in the right adrenal gland area	CS at 38 weeks	CHTH<br>metastases and masses regressed after 4 months
4.	Chapa et al. 2017 [15]	1	neuroblastoma	37	polyhydramnios, decreased foetal movement, pathologic liver mass	emergency CS at 37 weeks	CHTH<br>died on the 46th day after the delivery
5.	Izbizky et al. 2005 [16]	1	foetal bilateral adrenal carcinoma	26	polyhydramnios, bilateral multicystic abdominal masses	pPROM - spontaneous VD at 26 weeks	died shortly after birth

6.	Cornette et al. 2009[17]	1	mesenchymal hamartoma of the liver	33	polyhydramnios	CS at 35 weeks	laparotomy on the 2nd day after the delivery<br>follow – up at 6 months – no recurrence
7.	van de Bor et al. 1989 [18]	1	hepatoblastoma	29	polyhydramnios	spontaneous VD at 31 weeks	died shortly after birth
8.	Vadeyar et al. 2000 [19]	1	nephroblastoma	28	polyhydramnios, subcutaneous oedema, pathologic mass in the left upper abdomen	amnioreduction, 3 hours after – emergency CS	died shortly after birth
9.	Holzgrewe et al. 1985 [20]	1	foetal ovarian cyst	37	polyhydramnios, foetal bowel obstruction	induced VD at 39 weeks	laparotomy on the day of delivery
10.	Shima et al. 2003 [21]	1	congenital fibrosarcoma	24	polyhydramnios, foetal ascites	CS at 34 weeks	laparotomy on the day of delivery
11.	Do et al. 2015 [22]	1	mesoblastic nephroma	32+1	polyhydramnios, homogeneous mass in the right kidney	pPROM, VD at 35+5 weeks	nephrectomy on the 3rd after the delivery<br>CHTH<br>8 months – no sign of recurrence
12.	Kato et al. 2022 [23]	1	mesoblastic nephroma	32+3	polyhydramnios, renal tumour	CS at 36+2 weeks	resuscitation after birth and emergency laparotomy<br>36 months – no signs of recurrence
13.	Al-Turkistani et al. 2008 [24]	1	mesoblastic nephroma	28	polyhydramnios	amnioreduction, CS at 30 weeks	4th week after the delivery – laparotomy and left nephrectomy
14.	Daskas et al. 2002 [25]	1	mesoblastic nephroma	33	polyhydramnios, pathologic renal mass	amnioreduction, emergency CS (placental	tumour resection<br>one year after the surgery – no signs of recurrence

						abruption) at 33 weeks	
15.	Che et al. 2021 [26]	1	mesoblastic nephroma	24	polyhydramnios, pathologic renal mass	induced VD at 38+5 weeks	nephrectomy on the 9th day after the delivery<br>6th month – no signs of recurrence
16.	Mata et al. 2019 [27]	1	mesoblastic nephroma	32	polyhydramnios, pathologic renal mass	amnioreduction, pPROM, CS at 34+5 weeks	10th day after the delivery nephroureterectomy<br>6th month of life signs of microcephaly, hypotonia and developmental delay
17.	Chen et al. 2003 [28]	1	mesoblastic nephroma	22+3	polyhydramnios, foetal hydrops	amnioreduction, pPROM at 25+2 weeks, VD	died shortly after delivery
18.	Chen et al. 2021 [29]	4	mesoblastic nephroma	1. 31<br>2. 32<br>3. 29<br>4. 31`	polyhydramnios, pathologic renal masses	1. CS at 32 weeks<br>2. CS at 35 weeks<br>3. CS at 31 weeks<br>4. VD at 35 weeks	1. hypertension; surgery<br>- 33 months of follow up – no signs of recurrence<br>2. hypertension; surgery<br>- 14 months of follow up – no signs of recurrence<br>3. hypertension; surgery<br>- 12 months of follow up – no signs of recurrence<br>4. hypertension; surgery<br>- 12 months of follow up – no signs of recurrence
19.	Blank et al. 1978[30]	3	mesoblastic nephroma	1. 32<br>2. 35<br>3. 36	polyhydramnios	1. amnioreduction, pPROM at 34 weeks, CS<br>2. CS<br>3. spontaneous VD	1. 4th day after the delivery – right nephrectomy<br>2. surgery, tear in the inferior vena cava – neonate died<br>3. 8th day after the delivery – nephrectomy
20.	Kimani et al. 2020 [31]	1	mesoblastic nephroma	33	polyhydramnios	pPROM at 34 weeks, VD	neonatal sepsis<br>on the 20th day after the delivery - laparoscopy
21.	Robertson-Bell et al. 2017 [32]	1	mesoblastic nephroma	32	polyhydramnios	amnioreduction, pPROM at 32 weeks, VD	hypertension, surgery

22.	Allen et al. [33]	1	ovarian cyst	33	polyhydramnios, anechoic cyst, compression of foetal bowel	35th week amnioreduction, elective CS at 38 weeks	3rd after the delivery – laparoscopy and cystectomy
23.	Caballes et al. 2020 [34]	1	immature gastric teratoma	36	polyhydramnios, foetal ascites, meconium peritonitis	emergency CS	28th day after the delivery - laparotomy with en bloc excision of a massive tumor and section of the greater curvature of the stomach
24.	Falik-Borenstein et. al 1991 [35]	1	immature gastric teratoma	18	polyhydramnios, placental overgrowth, abdominal wall defect	CS at 39 weeks	5th hour of life – surgery, partial gastrectomy
25.	Jeong et al. 2012 [36]	1	immature gastric teratoma	34	polyhydramnios, intraperitoneal calcifications, ascites, dilated bowel	elective CS at 36 weeks	emergency laparotomy – resection of the tumor
26.	Gupta et al. 2008 [37]	1	sacrococcygeal teratoma	21	polyhydramnios, subcutaneous oedema of the foetus, cardiomegaly	termination of pregnancy	NA
27.	Greenberg et al. 1986 [38]	1	bilateral nephroblastoma	31	polyhydramnios, foetal ascites, nephromegaly	amnioreduction VD at 37 weeks	8th month of life – laparotomy one year follow up – developmental delay

CS, Caesarean section; VD, vaginal delivery; GA, gestational age; CHTH, chemotherapy; pPROM, preterm premature rupture of membranes; NA, not applicable.
